# 375. Back to School Unequal: Neighborhood Disadvantage and Asthma-related Emergency Department Visits in School-Aged Children

**DOI:** 10.1093/ofid/ofae631.116

**Published:** 2025-01-29

**Authors:** Peter Dunphy, Elizabeth Matsui, Paul Rathouz, Sarah Chambliss, Rebecca Zárate, Susan Balcer-Whaley, Darlene Bhavnani

**Affiliations:** University of Texas at Austin, Austin, TX; University of Texas at Austin, Austin, TX; The University of Texas at Austin, Austin, Texas; The University of Texas at Austin Dell Medical School, Austin, Texas; The University of Texas at Austin, Austin, Texas; The University of Texas at Austin, Austin, Texas; Dell Medical School, University of Texas at Austin, Austin, Texas

## Abstract

**Background:**

Asthma-related emergency department (ED) visit rates among children in a typical year closely follow the school calendar, with a sharp increase in rates when students return to school. The well-known increase in asthma exacerbations at the start of the school year is largely attributable to increases in respiratory viral infection. Because neighborhood disadvantage is strongly tied to asthma exacerbations, we hypothesized that greater neighborhood disadvantage would be associated with a greater increase in the rate of asthma exacerbations after the start of school.

**Figure:**
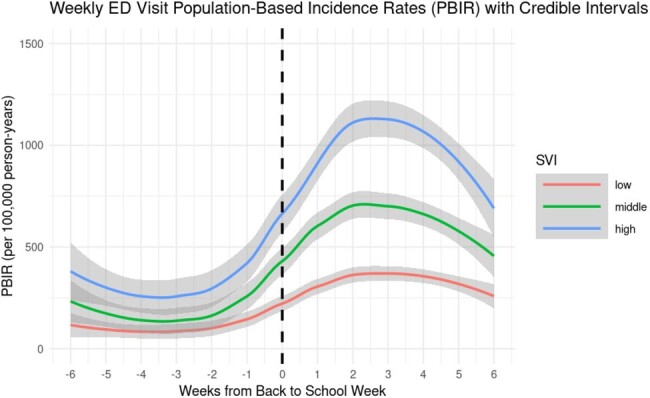

**Methods:**

We summarized Texas Health Care Information Collective data (2016-2019) on asthma-related ED visits among children (ages 5-17) in four Texas metropolitan statistical areas (Houston, Dallas, Austin, and San Antonio) by week and census tract. Neighborhood disadvantage was characterized at the census-tract level using tertiles of Social Vulnerability Index (SVI), percent uninsured, and percent living below the federal poverty line. Within each tertile, we described population-based incidence rates of asthma-related ED visits per 100,000 person-years in the 6 weeks before and after the week that school started. We also modeled weekly incidence rates and pointwise 95% confidence intervals by week using a locally weighted smoothing model.

**Figure:**
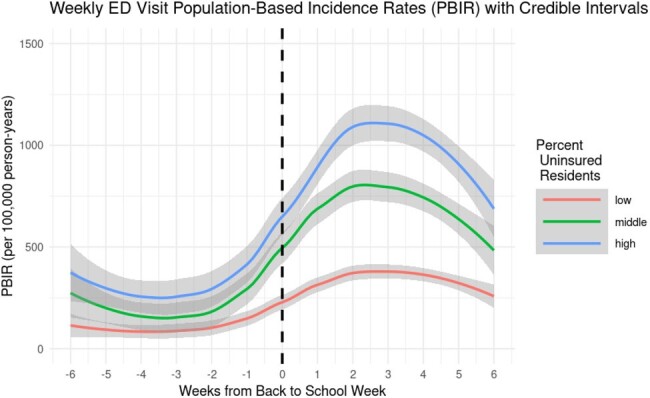

**Results:**

Comparing the 6-week periods before and after the week that school started, the incidence rate of asthma-related ED visits in high-, medium-, and low-SVI census tracts increased by 60, 39, and 20 cases per 100,000 person-years, respectively, representing a 3-fold difference between high- and low-SVI census tracts. Census tracts with high SVI had the greatest modeled increase in weekly rates of asthma-related ED visits after the start of school (Figure 1). Results were qualitatively similar for the percent of the population uninsured and below the federal poverty line (Figures 2-3).

**Figure:**
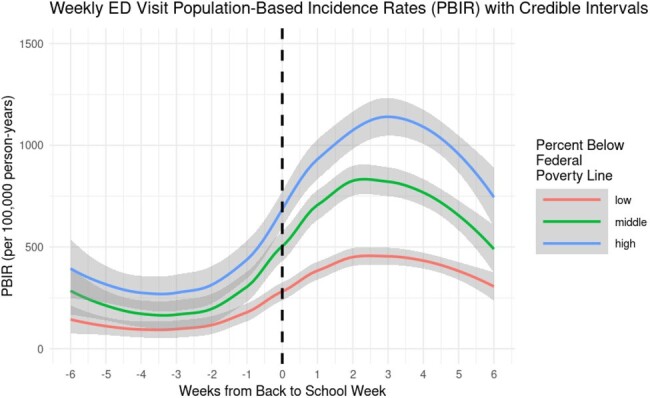

**Conclusion:**

Greater neighborhood disadvantage was associated with a greater increase in the rate of asthma related ED visits after the start of school, suggesting that neighborhood disadvantage may contribute to exacerbation risk by increasing the risk of respiratory virus infection.

**Disclosures:**

**Paul Rathouz, PhD**, Sunovion Pharmaceuticals: DSMB Member

